# Role of the Carotid Bodies in the Hypertensive and Natriuretic Responses to NaCl Load in Conscious Rats

**DOI:** 10.3389/fphys.2018.01690

**Published:** 2018-12-04

**Authors:** Elaine Fernanda da Silva, Aryanne Batista Soares de Melo, Eulício de Oliveira Lobo Júnior, Karla Lima Rodrigues, Lara Marques Naves, Wendell Karlos Tomazelli Coltro, Ana Cristina Silva Rebelo, André Henrique Freiria-Oliveira, José Vanderlei Menani, Gustavo Rodrigues Pedrino, Eduardo Colombari

**Affiliations:** ^1^Department of Physiology and Pathology, School of Dentistry, São Paulo State University, Araraquara, Brazil; ^2^Department of Physiological Sciences, Biological Sciences Institute, Federal University of Goiâs, Goiânia, Brazil; ^3^Chemistry Institute, Federal University of Goiâs, Goiânia, Brazil; ^4^Department of Morphology, Biological Sciences Institute, Federal University of Goiâs, Goiânia, Brazil

**Keywords:** carotid afferents, blood pressure, sodium balance, hypernatremia, high salt intake, fluid-electrolyte control

## Abstract

Hyperosmotic challenges trigger a hypertensive response and natriuresis mediated by central and peripheral sensors. Here, we evaluated the importance of the carotid bodies for the hypertensive and natriuretic responses to acute and sub-chronic NaCl load in conscious rats. Male Wistar rats (250–330 g) submitted to bilateral carotid body removal (CBX) or sham surgery were used. One day after the surgery, the changes in arterial blood pressure (*n* = 6–7/group) and renal sodium excretion (*n* = 10/group) to intravenous infusion of 3 M NaCl (1.8 mL/kg b.w. during 1 min) were evaluated in non-anesthetized rats. Another cohort of sham (*n* = 8) and CBX rats (*n* = 6) had access to 0.3 M NaCl as the only source of fluid to drink for 7 days while ingestion and renal excretion were monitored daily. The sodium balance was calculated as the difference between sodium infused/ingested and excreted. CBX reduced the hypertensive (8 ± 2 mmHg, vs. sham rats: 19 ± 2 mmHg; *p* < 0.05) and natriuretic responses (1.33 ± 0.13 mmol/90 min, vs. sham: 1.81 ± 0.11 mmol/90 min; *p* < 0.05) to acute intravenous infusion of 3 M NaCl, leading to an increase of sodium balance (0.38 ± 0.11 mmol/90 min, vs. sham: -0.06 ± 0.10 mmol/90 min; *p* < 0.05). In CBX rats, sub-chronic NaCl load with 0.3 M NaCl to drink for 7 days increased sodium balance (18.13 ± 4.45 mmol, vs. sham: 5.58 ± 1.71 mmol; *p* < 0.05) and plasma sodium concentration (164 ± 5 mmol/L, vs. sham: 140 ± 7 mmol/L; *p* < 0.05), without changing arterial pressure (121 ± 9 mmHg, vs. sham: 116 ± 2 mmHg). These results suggest that carotid bodies are important for the maintenance of the hypertensive response to acute hypertonic challenges and for sodium excretion to both acute and chronic NaCl load.

## Introduction

In order to maintain body fluid homeostasis, behavioral, neuroendocrine, and cardiovascular adjustments are triggered during hyperosmotic challenges (Fitzsimons, 1998; Antunes-Rodrigues et al., 2004; Antunes et al., 2006). Acute NaCl load increases arterial pressure and renal sodium excretion (Hatzinikolaou et al., 1981; McKinley et al., 1992; Schoorlemmer et al., 2000; Antunes et al., 2006; Silva et al., 2013). The increase in arterial pressure in response to hypertonic NaCl infused intravenously is a consequence of the elevation of total peripheral resistance (Garcia-Estan et al., 1989) due to a vasoconstrictor effect mediated by both sympathetic nervous system and vasopressinergic mechanisms (Antunes et al., 2006). Regarding the sympathetic activity, it is important note that intravenous hypertonic NaCl produces different regional effects with a marked increase in lumbar sympathetic nervous activity (SNA) and reduction in renal SNA (Weiss et al., 1996). Although the beneficial effects of the pressor response to acute NaCl load is not fully understood, the increase in arterial pressure may elevate the tissue perfusion, including for the kidneys, which contributes to enhance glomerular filtration rate and urinary sodium (Na^+^) loss. In addition, hormonal and autonomic mechanisms also affect natriuresis. Oxytocin released in response to high Na^+^ plasma induces natriuresis (Landgraf et al., 1988; Huang et al., 1995) and the reduction in renal sympathetic activity (Weiss et al., 1996; Pedrino et al., 2008) also contributes to the renal vasodilation (Pedrino et al., 2008) and natriuresis (Johns et al., 2011).

Specializing cells located in the forebrain areas, such as the subfornical organ (SFO) and the region surrounding the anteroventral third ventricle (AV3V), are activated by extracellular osmolality intrinsically (Anderson et al., 2000; Kinsman et al., 2017) and signaling for cardiovascular and renal responses during hyperosmotic challenges (Bourque et al., 1994). Lesions of the SFO and AV3V reduced the pressor and natriuretic responses to acute NaCl load (Thornborough et al., 1973; McKinley et al., 1992; Pedrino et al., 2005). Moreover, it is also possible that additional information from portal-hepatic osmoreceptors and cardiovascular afferents participate in the natriuretic response to elevate Na^+^ levels (Morris and Alexander, 1988; Ishiki et al., 1991; Morita et al., 1995; Pedrino et al., 2016). The signals from peripheral sensors might be integrated in the brain areas involved in the fluid-electrolyte control and cardiovascular regulation (Yamashita, 1977; Ciriello and Calaresu, 1980; Spyer, 1981) contributing for osmoregulation. In this direction, Na^+^ homeostasis appears to be controlled by a series of central and peripheral sensors triggering osmoregulatory mechanisms.

The carotid bodies contain chemosensitive cells that play an important role monitoring arterial blood oxygen levels and triggering reflexes to maintain homeostasis during hypoxemia (reviewed by López-Barneo et al., 2016). In addition to the short-term monitoring of blood gas tension, carotid bodies are also sensitive to low glucose, pro-inflammatory cytokines, angiotensin II, adenosine, and changes in the osmolality (Trzebski et al., 1978; Gallego et al., 1979; Runold et al., 1990; Allen, 1998; Pardal and López-Barneo, 2002; Zhang et al., 2007; Liu et al., 2009), evidencing the multisensorial nature of the carotid bodies. Previous studies demonstrated that hyperosmotic solutions increase the frequency of discharge of the carotid body afferent fibers *in vivo* (anesthetized cats) (Trzebski et al., 1978) as well as *in vitro* (Gallego et al., 1979). A study from our laboratory showed that carotid chemoreceptors integrity is important for the restoration of arterial blood pressure induced by intravenous infusion of hypertonic NaCl in rats submitted to hypotensive hemorrhage (Pedrino et al., 2011). Recently, we showed that carotid bodies inactivation prevented the renal vasodilation and sympathoinhibition to acute NaCl load in anesthetized rats (Pedrino et al., 2016). However, whether carotid bodies participate in the regulation of arterial pressure and effectively contribute for renal sodium excretion during acute and subchronic NaCl load in conscious rats have not been studied yet.

In the current study, we investigated the importance of carotid bodies for the natriuresis and hypertensive response to acute and sub-chronic NaCl load in non-anesthetized, conscious freely moving rats. Renal sodium excretion and arterial pressure were measured in rats with either CBX or sham surgery that received an acute intravenous infusion of 3 M NaCl or had access to 0.3 M NaCl, instead of water, to drink for 1 week.

## Materials and Methods

### Animals

Male Wistar rats weighing 250–330 g were used. Animals were housed in a temperature-controlled room (22–24°C) with a 12:12-h light-dark cycle (lights on at 06:00 AM) and had continuous access to standard rat chow and either tap water or 0.3 M NaCl as their only source of fluid. Experimental protocols were approved by the Ethics Committee for Animal Use from Federal University of Goiás (number 034/12).

### Carotid Body Removal

The bilateral removal of carotid bodies was made using a modification of the technique described by Abdala et al. (2012). Briefly, rats were anesthetized with halothane (2% in O_2_) and fixed in supine position. A midline incision (3 cm) was made in the neck, to expose the muscles that cover the trachea and the region of the carotid artery bifurcation. After dissection of sternocleidomastoid muscle, the carotid bifurcation was exposed, the occipital artery was retracted and the carotid body visualized, carefully isolated and removed using a surgical microscope. Sham-denervated rats were submitted at a midline incision (3 cm) in the neck and dissection of sternocleidomastoid muscle.

### Arterial Pressure and Heart Rate Recordings

Animals were anesthetized with halothane (2% in O_2_) and a catheter (PE-10 connected to PE-50 tubing, CPL Medicals, São Paulo, SP, Brazil) was inserted into the abdominal aorta through the right femoral artery for arterial pressure measurement. The right femoral vein was catheterized for drug administration [phenylephrine and potassium cyanide(KCN)] and hypertonic NaCl infusion. Catheters were tunneled subcutaneously and exteriorized between the scapulas to record arterial pressure and HR non-anesthetized freely moving rat. For this, the arterial catheter was connected to a pressure transducer (MLT 0380, ADInstruments, Colorado Springs, United States) attached to an amplifier (Bridge Amplifier ETH-200, CB Sciences, Dover, NH, United States). The PAP signals were acquired by a data acquisition system (PowerLab System 8/25, ADInstruments, Colorado Springs, CO, United States) and recorded in a computer using appropriate software (Chart Pro v.7.3.1., ADInstruments, Colorado Springs, CO, United States), at a sampling frequency of 2 kHz. The MAP and HR were calculated from PAP signals.

### Baroreflex and Chemoreflex Tests

The changes in the arterial pressure and HR produced by baroreflex and chemoreflex activation were tested with the intravenous infusion of phenylephrine (α_1_ adrenergic agonist, Sigma-Aldrich, St Louis, MO, United States, 1, 1.5, and 2 μg in 100 μL) and KCN (40 μg in 100 μL; Sigma-Aldrich, St, Louis, MO, United States), respectively. At least 5 min were allowed between each injection or until cardiovascular parameters returned to baseline. Animals included in the CBX group were those in which the bradycardia and hypertensive responses to peripheral chemoreceptor activation with KCN were completely abolished, without changes in the baroreflex-induced bradycardia (to an increase of 25–40 mmHg in the arterial pressure). The changes produced by chemoreflex activation in the MAP and HR were determined as the difference from baseline. The baroreflex index was calculated as a ratio from maximum variations in HR and MAP induced by phenylephrine infusion.

### Acute Sodium Load

Rats had continuous daily access to food and tap water. On the day of the test, 3 M NaCl (1.8 mL/kg of body weight during 60 s) was infused through the catheter implanted in the right femoral vein. During the hyperosmotic test, animals had no access to food and water.

### Sub-Chronic Sodium Load

Rats were housed individually in metabolic cages with free access to food and 0.3 M NaCl as their only source of fluid to drink for 7 days. Hypertonic NaCl was provided from burettes fitted with metal drinking spouts.

### Plasma and Urinary Analysis

Rats were housed individually in metabolic cages. Urine samples were collected after a period of 24 h for acclimatizing. The urinary volume was quantified and urinary sodium concentration was measured using a flame photometer (400 Flame Photometer; Corning; currently marketed by Sherwood Scientific, Cambridge, United Kingdom). The amount of sodium in the urine was determined by calculating the product of the urinary volume and sodium concentration. The sodium balance was calculated as the difference between the amounts of sodium infused/ingested and excreted.

Blood samples from sham and CBX rats were collected before the sub-chronic NaCl load (when the rats had water to drink) and at the end of the 7 days of access to NaCl. Rats were anesthetized with halothane (2% in O_2_) and had the tail end cut for blood collection.

### Experimental Protocols

Three experimental protocols were performed. In the first protocol, the effects of CBX on the cardiovascular responses to acute NaCl load were evaluated. Briefly, rats were anesthetized with halothane and submitted to CBX or sham surgery followed by catheterization of the femoral artery and vein. After recovery from anesthesia (∼4 h), when the rats had return to move freely, baro- and chemoreflex tests were performed to confirm the selectivity of CBX. One day after the surgery (∼24 h), sham-denervated (*n* = 7) and CBX (*n* = 6) rats received intravenous infusion of 3 M NaCl (1.8 mL/kg b.w. during 1 min) while arterial pressure and HR were recorded. The cardiovascular parameters were evaluated for 30 min after the infusion.

In the second protocol, another group of rats were used to evaluate the effects of CBX on renal sodium excretion induced by acute NaCl load. Briefly, rats were anesthetized with halothane and submitted to CBX or sham surgery. After recovery of the surgical procedure (∼2 days), rats had a catheter inserted into the femoral vein. Sham-denervated (*n* = 10) and CBX rats (*n* = 10) were housed individually in metabolic cages during the period post-surgical for acclimatizing, as described before (Silva et al., 2013). After, animals received intravenous infusion of 3 M NaCl (1.8 mL/kg b.w. during 1 min) and urine samples were collected at 10, 30, 60, and 90 min after hypertonic NaCl infusion. The urine was collected by gravity in polypropylene tubes, and urinary volume was measured using a graduated glass cylinder with 0.1 mL divisions. On the day after the hyperosmotic test, rats were anesthetized, and a catheter was implanted in the abdominal aorta through right femoral artery. Baro- and chemoreflex tests were performed while arterial pressure and HR were recorded around 24 h after the artery cannulation.

In the third protocol, the effects of the sub-chronic NaCl load on renal sodium excretion, arterial pressure, and HR were investigated. Sham and CBX surgery were performed as described above. After water and food intake returned to pre-surgical levels (∼2 days), sham-denervated (*n* = 8), and CBX rats (*n* = 6) were housed individually in metabolic cages for a period of 24 h for acclimatizing with free access to standard rat chow and water. After this period, the water and food consumption was monitored for 2 days. Following, water was removed from the cages and rats received 0.3 M NaCl to drink and standard chow for consecutive 7 days. Water (in the first 2 days), food and hypertonic NaCl intake and urine excretion were measured daily. Water and hypertonic NaCl were measured using graduated burettes fitted with metal drinking spouts. The Na^+^ ingested through the food intake was calculated. The urine was collected by gravity in beakers. At the end of the ingestion and renal excretion tests, rats were anesthetized with halothane and catheters were implanted in the abdominal aorta through right femoral artery and femoral vein. Arterial pressure and HR were recorded and baro- and chemoreflex tests were performed around 24 h after the surgery. Rats were maintained with hypertonic fluid until the end of experimentation.

At the end of each experimental protocol, rats were killed by overdose with anesthetic (sodium pentobarbital, 60 mg/kg of body weight) administered intraperitoneally, followed by an incision in the diaphragm muscle.

### Data Analysis

Results were expressed as means ± SEM. Statistical analyses were performed using SigmaPlot (v13; Systat Software Inc., San Jose, CA, United States). Two-way ANOVA followed by Newman–Keuls’s post-test was used to analyze the changes in MAP and HR and renal sodium excretion in response to acute NaCl load, as well as food and hypertonic NaCl intake, renal sodium output, and plasma sodium concentration to sub-chronic NaCl load. Unpaired Student’s t test was used to analyze the baseline levels of MAP and HR and responses evoked during baro- and chemoreflex tests between the groups. The statistical test applied in each case is indicated in the corresponding figure legend. Significance was considered at *p* < 0.05.

## Results

### Confirmation of CBX Efficacy

The changes in MAP and HR produced by chemoreflex activation with i.v. KCN in sham rats that received acute NaCl load (39 ± 3 mmHg and -126 ± 16 bpm, respectively) were abolished in CBX rats that received acute NaCl load (7 ± 2 mmHg and 16 ± 2 bpm, respectively, compared to sham; *p*<0.05). Similarly, the changes in MAP and HR produced by i.v. KCN in sham rats that ingested 0.3 M NaCl (52 ± 5 mmHg and -150 ± 19 bpm, respectively) were abolished in CBX rats that ingested 0.3 M NaCl (-7 ± 3 mmHg and 17 ± 7 bpm, respectively, compared to sham; *p*<0.05). These results confirm the efficacy of CBX.

Baroreflex responses to i.v. phenylephrine infusion did not differ between sham and CBX rats that were submitted to acute NaCl load (sham rats: -2.00 ± 0.13 Δbpm/ΔmmHg, compared to CBX: -1.75 ± 0.16 Δbpm/ΔmmHg; *p* > 0.05). Baroreflex index was also similar between sham and CBX rats that ingested 0.3 M NaCl (sham: -2.56 ± 0.32 Δbpm/ΔmmHg; compared to CBX: -1.79 ± 0.22 Δbpm/ΔmmHg; *p* > 0.05).

### Changes in Arterial Pressure, Heart Rate, and Renal Sodium Excretion to Acute NaCl Load in CBX Rats

Baseline values of MAP and HR were not different between sham (107 ± 4 mmHg and 331 ± 9 bpm, respectively) and CBX (98 ± 5 mmHg and 330 ± 8 bpm, respectively) non-anesthetized rats. Body weights were also similar in sham (308 ± 8 g) and CBX rats (293 ± 9 g).

**FIGURE 1:**
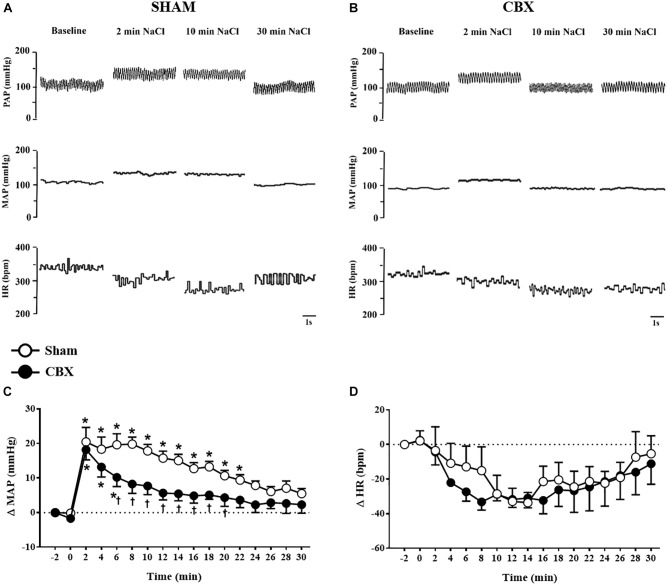
**(A,B)** Pulsatile arterial pressure (PAP), mean arterial pressure (MAP), and heart rate (HR) recordings from non-anesthetized sham and carotid body removal (CBX) rats, respectively, during baseline and 2, 10, and 30 min after the infusion of 3 M NaCl (1.8 mL/kg body weight, over 1 min). **(C,D)** Changes in mean arterial pressure (ΔMAP) and heart rate (ΔHR), respectively, produced by 3 M NaCl infusion in sham (*n* = 7) and CBX (*n* = 6) rats. Data in **(C,D)** were analyzed by two-way ANOVA followed by Newman–Keuls’s post-test. ^∗^Different from pre-infusion (0 min) in the same group. ^†^Different from sham rats (*p* < 0.05).

Intravenous infusion of 3 M NaCl (1.8 mL/kg of body weight) in sham rats produced pressor response that reached a peak (20 ± 4 mmHg) 2 min after starting the infusion (Figures 1A,C). This response slowly returned to baseline pre-infusion level reaching baseline at *t* = 24 min. In CBX rats, the pressor response also reached a similar peak (18 ± 3 mmHg) 2 min after starting the infusion of hypertonic NaCl, however returned to baseline pre-infusion level faster (*t* = 8 min) (CBX: 8 ± 2 mmHg, vs. sham rats: 19 ± 2 mmHg) [*F*(1,187) = 66.94, *p <* 0.05] (Figures 1B,C).

Acute hypertonic NaCl produced no significant changes on HR in sham (-32 ± 4 bpm, 12 min after the infusion) and CBX rats (-31 ± 5 bpm, 12 min after the infusion), [*F*(1,187) = 1.85, *p >* 0.05] (Figures 1A,B,D).

**FIGURE 2:**
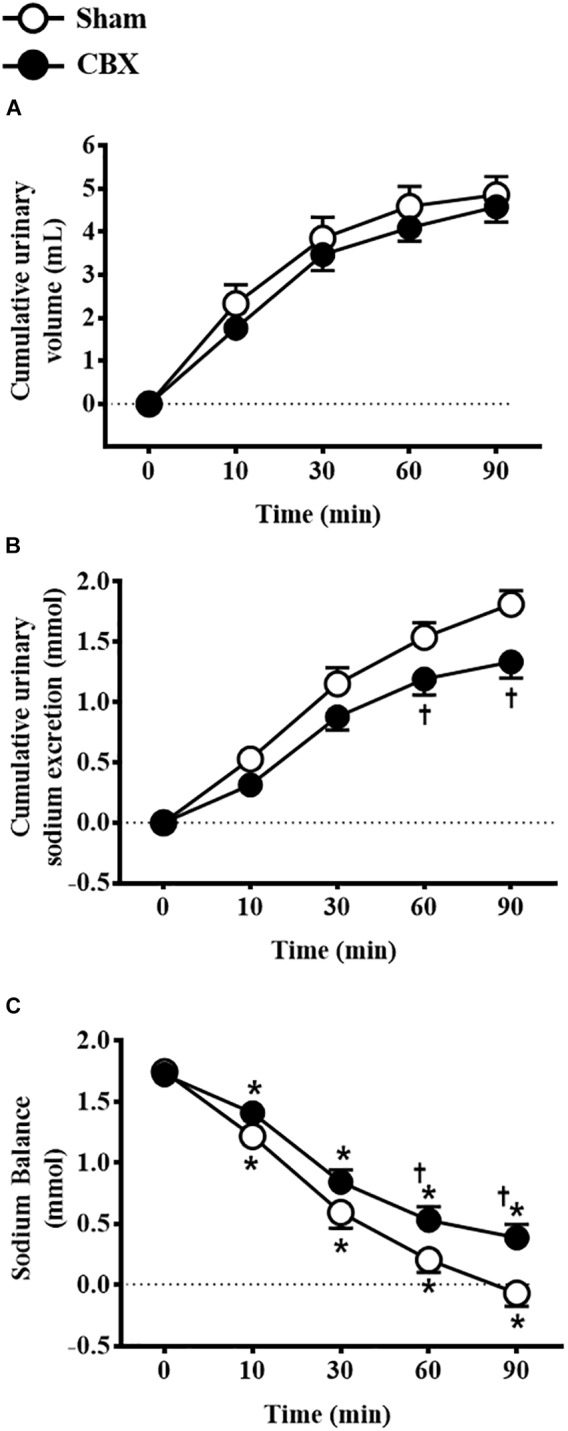
Cumulative **(A)** urinary volume and **(B)** urinary sodium excretion and **(C)** sodium balance in sham (*n* = 10) and carotid body removal (CBX, *n* = 10) rats that received intravenous infusion of 3 M NaCl (1.8 mL/kg body weight, over 1 min). Data were analyzed by two-way ANOVA followed by Newman–Keuls’s post-test. In **(C)**, ^∗^different from time 0. In **(B,C)**, ^†^different from sham rats (*p* < 0.05).

Intravenous infusion of 3 M NaCl similarly increased urinary volume in sham (4.9 ± 0.4 mL/90 min) and CBX rats (4.5 ± 0.3 mL/90 min) [*F*(1,90) = 2.38, *p >* 0.05] (Figure 2A). However, CBX reduced urinary sodium excretion (CBX: 1.33 ± 0.13 mmol/90 min, vs. sham: 1.81 ± 0.11 mmol/90 min) [*F*(1,90) = 16.59, *p <* 0.05] and increased sodium balance (CBX: 0.38 ± 0.11 mmol, vs. sham: -0.06 ± 0.10 mmol, 90 min after hypertonic NaCl infusion) [*F*(1,90) = 15.93, *p <* 0.05] (Figures 2B,C). Sham rats excreted 100 ± 6.4% of the sodium infused, whereas CBX rats excreted only 77 ± 6.7% of sodium until 90 min after hypertonic NaCl infusion. These results indicate a reduced capacity of CBX rats to excrete sodium after an acute NaCl overload.

### Daily Sodium Intake and Renal Excretion During Sub-Chronic NaCl Load

**FIGURE 3:**
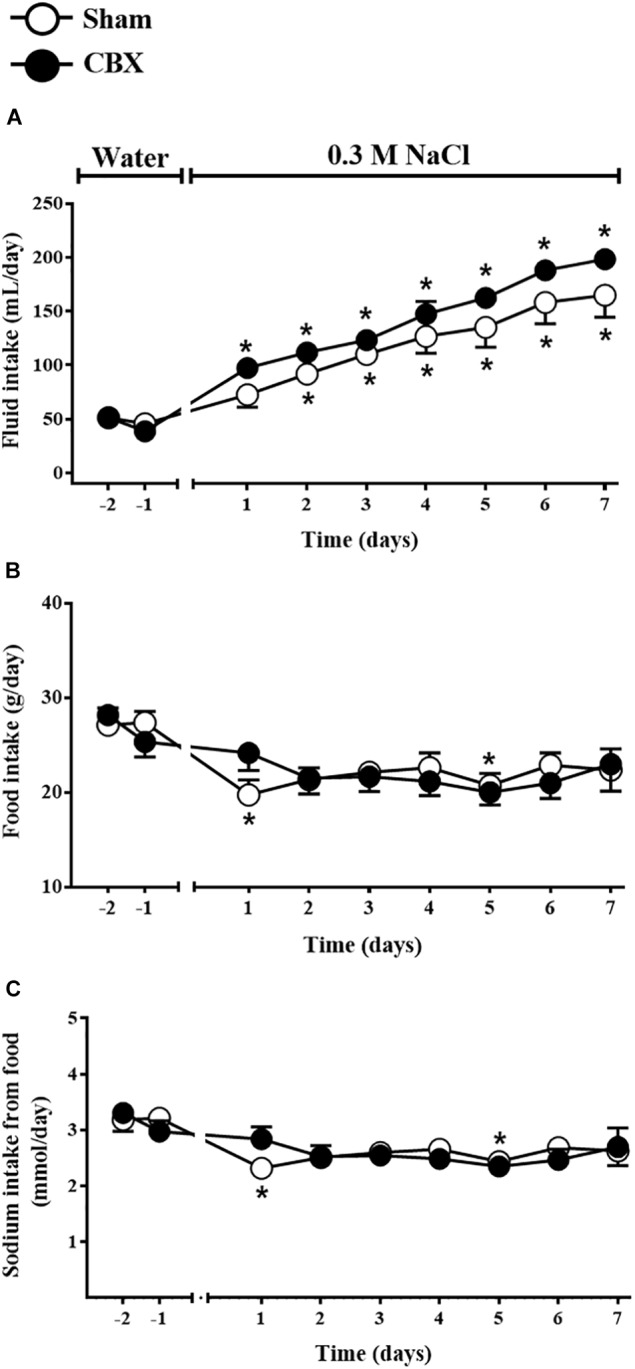
Daily **(A)** fluid and **(B)** food intake and **(C)** sodium intake from the food by sham (*n* = 8) and carotid body removal (CBX, *n* = 6) rats during access to water or 0.3 M NaCl to ingest. Data were analyzed by two-way ANOVA followed by Newman–Keuls’s post-test. ^∗^Different from the period of access to water (*p* < 0.05).

The sub-chronic NaCl load was promoted by the access to the 0.3 M NaCl as the only source of fluid to drink for 7 days. Daily 0.3 M NaCl intake gradually and similarly increased in sham rats from the second day of treatment (91 ± 7 mL/day, vs. pre-load: 47 ± 5 mL/day of water) until the last day of the test (164 ± 20 mL/day) and in CBX animals from the first day of treatment (97 ± 9 mL/day, vs. pre-load: 38 ± 2 mL/day of water) until the last day of the test (198 ± 4 mL/day) [*F*(1,108) = 9.72, *p <* 0.05] (Figure 3A). Daily food intake was not different between sham and CBX rats, however, in sham rats food intake was reduced in the first day of treatment (19 ± 1 g/day, vs. pre-load: 28 ± 1 g/day) [*F*(1,108) = 0.003, *p <* 0.05], but not in CBX rats (24 ± 1 g/day, vs. pre-load: 25 ± 1 g/day) (Figure 3B). The sodium consumed through the food did not differ between sham and CBX rats, but sham rats reduced the sodium intake in the first day of treatment (2.31 ± 0.18 mmol/day, vs. pre-load: 3.18 ± 0.20 mmol/day) [*F*(1,108) = 0.003, *p <* 0.05] (Figure 3C).

**FIGURE 4:**
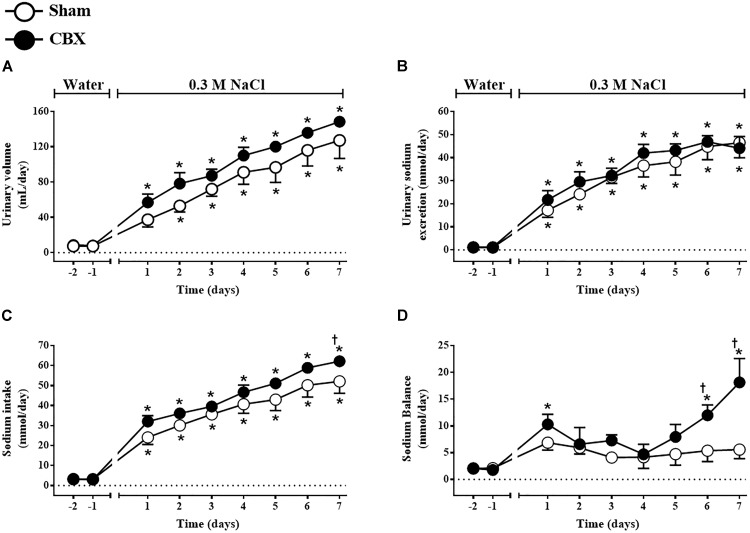
Daily **(A)** urinary volume, **(B)** urinary sodium excretion, **(C)** sodium intake, and **(D)** sodium balance in sham (*n* = 8) and carotid body removal (CBX, *n* = 6) rats during access to water or 0.3 M NaCl to ingest. Data were analyzed by two-way ANOVA followed by Newman–Keuls’s post-test. ^∗^Different from the period of access to water. ^†^Different from sham rats (*p* < 0.05).

Daily urinary volume increased in sham rats from the second day of treatment (53 ± 6 mL/day, vs. pre-load: 8 ± 2 mL/day) until the last day of the test (127 ± 20 mL/day) and also in CBX rats from the first day of treatment (57 ± 9 mL/day, vs. pre-load: 8 ± 2 mL/day) until the last day of the test (149 ± 4 mL/day) [*F*(1,108) = 9.39, *p <* 0.05] (Figure 4A). Daily urinary sodium excretion also gradually and similarly increased in sham (46.51 ± 6.55 mmol/day, in the last day of the test, vs. pre-load: 1.10 ± 0.20 mmol/day) and CBX rats (44.07 ± 5.12 mmol/day, in the last day of the test, vs. pre-load: 1.21 ± 0.38 mmol/day) [*F*(1,108) = 1.51, *p <* 0.05] (Figure 4B). The total daily sodium intake gradually increased in sham (52.08 ± 5.98 mmol/day, in the last day of the test, vs. pre-load: 3.21 ± 0.13 mmol/day) and in CBX rats (62.20 ± 1.19 mmol/day, in the last day of the test, vs. pre-load: 2.97 ± 0.18 mmol/day), with a higher intake in CBX rats in the last day of test [*F*(1,108) = 11.21, *p <* 0.05] (Figure 4C). Sodium balance increased in CBX rats in the last two days of test (18.13 ± 4.45 mmol/day, vs. sham rats: 5.58 ± 1.71 mmol/day in the last day of test) [*F*(1,108) = 14.52, *p <* 0.05] (Figure 4D). At the end of the test sham rats excreted in the urine 90 ± 4.0% of all sodium ingested, whereas CBX rats excreted only 70 ± 7.5%, indicating a reduced capacity of CBX rats to excrete all sodium ingested.

Plasma sodium concentration was similar between sham (142 ± 4 mmol/L) and CBX rats (147 ± 4 mmol/L) before the sub-chronic hyperosmotic challenge, however, increased in CBX rats at the end of the test (164 ± 5 mmol/L, vs. sham: 140 ± 7 mmol/L) [*F*(1,14) = 1.90, *p* < 0.05].

### Arterial Pressure and Heart Rate at the End of Sub-Chronic NaCl Load

At the end of 7 days of access to 0.3 M NaCl, MAP, and HR were not different between sham (116 ± 2 mmHg and 356 ± 6 bpm, respectively) and CBX rats (121 ± 9 mmHg and 377 ± 12 bpm, respectively).

## Discussion

The present results show that CBX reduced the hypertensive and natriuretic responses to acute intravenous infusion of hypertonic NaCl. CBX did not affect the increase in renal sodium excretion to sub-chronic NaCl load, but caused an increased NaCl ingestion resulting in an increase in sodium balance and plasma sodium concentration at the end of the 7 days of access to NaCl, without changing resting arterial blood pressure and HR. Therefore, the present results suggest for the first time that carotid bodies are important for the maintenance of the hypertensive response to acute NaCl load in non-anesthetized rats and are also part of the reflex pathway activated to regulate sodium balance during both acute and sub-chronic NaCl load. The present results together with previous studies showing that carotid bodies mediate sympathetic adjustments induced by hypernatremia in anesthetized rats (Silva et al., 2015; Pedrino et al., 2016), suggest that signals from carotid bodies modulate cardiovascular and fluid-electrolyte responses during salt overload.

Intravenous infusion of NaCl in the same concentration and dose (3 M NaCl, 1.8 mL/kg of body weight) has been used to produce a sudden increase in the plasma sodium concentration, without changing blood volume (Pedrino et al., 2006, 2016; Silva et al., 2013, 2015). Hypertonic NaCl infused intravenously in a concentration that produces hypernatremia triggers a sustained hypertensive response in intact rats (Pedrino et al., 2005; Silva et al., 2013). This pressor response has been described to depend on a vasoconstrictor effect mediated by sympathetic and vasopressinergic mechanisms (Hatzinikolaou et al., 1981; Garcia-Estan et al., 1989; Antunes et al., 2006). In this regards, Antunes et al. (2006) using conscious rats demonstrated that pre-treatment with prazosin (α_1_-adrenoceptor antagonist) produced an initial hypotension to intravenous NaCl followed by a pressor effect, whereas V_1a_ vasopressin receptor antagonist blocked the increase in MAP at any time point. Thus, it is proposed that the initial pressor effect elicited by intravenous hypertonic NaCl in conscious rats depends on both sympathetic and vasopressinergic mechanisms, whereas the prolonged increase in the arterial pressure is mediated mainly by vascular V_1a_ receptor activation (Antunes et al., 2006).

In the present study, the removal of carotid bodies attenuated the hypertensive response induced by intravenous infusion of hypertonic NaCl, suggesting that carotid bodies are involved in the hemodynamic adjustments during acute hypernatremia in conscious rats. Studies have reported that peripheral chemoreceptors stimulation was associated with activation of neurons in the supraoptic (SON) and hypothalamic paraventricular (PVN) nuclei (Yamashita, 1977; Berquin et al., 2000; Coldren et al., 2017). Moreover, the hypoxemia increased the neurohypophyseal blood flow and plasma vasopressin concentration, which are mediated by peripheral chemoreceptors (Wilson et al., 1987). Therefore, it is possible that the reduction in the hypertensive response to acute infusion of hypertonic NaCl in CBX rats might be a consequence of an impairment of vasopressinergic component.

Conscious sham rats that were infused intravenously with hypertonic NaCl excreted all sodium administered. Moreover, these animals presented a negative sodium balance at the end of the acute test, which could be produced by a possible salt storage in the skin interstitial fluid (Machnik et al., 2009; Kirabo, 2017; Nikpey et al., 2017). In these regards, a considerable amount of Na^+^ may be removed from the body and the skin might serve as major Na^+^ reservoir during salt overload (Nikpey et al., 2017). By measuring Na^+^ intake and excretion, we found no significant difference in the sodium balance in sham rats during sub-chronic salt load (those that had only 0.3 M NaCl to drink) compared to water intake. On the other hand, CBX rats presented a reduced capacity to excrete all sodium infused or ingested. In these animals the capacity to regulate sodium balance during hyperosmotic challenges was impaired, i.e., without the carotid bodies sodium balance was positive suggesting sodium retention in the body. Therefore, these new findings highlight by the first time that carotid bodies are involved in control of sodium balance during acute and sub-chronic NaCl load in conscious rats.

CBX rats ingested a higher amount of sodium in the seventh day of the test, whereas sodium excretion was similar in both sham and CBX groups. Thus, a possible imbalance between sodium ingestion and excretion resulted in significant positive sodium balance and increased levels of plasma sodium at the end of the hypertonic NaCl intake period. The tendency of CBX rats to retain sodium in the body is also confirmed by the increase in plasma sodium concentration in these animals. These results suggest that carotid bodies are involved in the mechanisms that control renal sodium excretion during enhanced NaCl load. Sinoaortic denervation (that removes both baro and chemoreceptor afferents) impaired the ability to control sodium balance in Dahl-salt sensitive rats fed with a standard-salt diet (Chen et al., 1998) and normotensive salt-resistant rats treated with high dietary salt (Dibona and Sawin, 2002). Here, using a technique for specific removal of carotid bodies without affecting the baroreceptor reflex, it is shown the importance of carotid bodies for the increase in the renal sodium excretion during hypertonic challenges.

It is possible that peripheral sensors are connected to hormonal and autonomic mechanisms involved in the natriuresis evoked by NaCl load (Morris and Alexander, 1988; Ishiki et al., 1991; Morita et al., 1995). Sinoaortic denervation reduced the baseline levels of atrial natriuretic peptide and the release of this hormone induced by hypertonic NaCl (Morris and Alexander, 1988). Recently, we showed that either sinoaortic denervation or selective inactivation of carotid body chemoreceptors abolished the renal vasodilation and sympathoinhibition induced by intravenous hypertonic NaCl infusion in anesthetized rats (Silva et al., 2015; Pedrino et al., 2016), which are important mechanisms to produce renal sodium excretion (Johns et al., 2011). Therefore, inability in reducing the renal sympathetic outflow in response to NaCl load might impairs the sodium excretion in CBX rats. Moreover, the renal sodium excretion has been linked to arterial pressure levels (for review Bie and Evans, 2017). In this regards, the pressure-natriuresis relationship describes sodium excretion rate as a function of arterial blood pressure (Guyton et al., 1972). Following this rule, the reduced pressor response to acute NaCl infusion found in CBX rats might impair sodium excretion. Similarly, in CBX rats that ingested 0.3 M NaCl, the short-time period of salt loading failed to produce arterial hypertension affecting sodium excretion, which resulted in a positive sodium balance. Thus, in these animals, the inability to increase the blood pressure during high NaCl intake resulted in an impaired capacity to excrete all sodium ingested and consequent sodium retention.

The involvement of central osmorreceptors in the natriuretic response to NaCl load is strongly supported by observations that lesions in the lamina terminalis abolished the renal sodium output to direct stimulation of the brain through intracarotid hypertonic NaCl administration in anesthetized cats (Thornborough et al., 1973) and also caused a large impairment when hypertonic NaCl was infused intravenously in conscious sheep (McKinley et al., 1992). The present results show that CBX reduced sodium excretion to acute intravenous NaCl infusion in about 23%, whereas the excretion during sub-chronic challenge was impaired in 30% in conscious CBX rats. These findings suggest that carotid bodies may send signals to central areas contributing to the full regulation of natriuretic mechanisms. Signals from carotid bodies gain access to the central nervous system through the NTS (Spyer, 1981). In turn, NTS neurons send projections to brainstem and hypothalamic areas involved in the hormonal and cardiovascular control (Ricardo and Koh, 1978; Sawchenko and Swanson, 1982; Bailey et al., 2006), such as the CVLM, PVN, and SON. Similar to which occurred in CBX rats, lesions of the A1 noradrenergic neurons in the CVLM attenuated the hypertensive response and natriuresis induced by hypertonic NaCl infusion in non-anesthetized rats (Silva et al., 2013). Therefore, like A1 noradrenergic neurons, carotid bodies also are important for pressor effects and natriuresis to acute NaCl load. Signals from carotid bodies might be processed in the brain and integrate the neuronal pathways involved in the cardiovascular and renal adjustments to hypertonic NaCl load.

## Conclusion

The present study shows that CBX affects cardiovascular response and sodium balance during NaCl load. Specifically, the results demonstrated that bilateral removal of carotid bodies shortened the hypertensive response and attenuated natriuresis induced by acute intravenous infusion of hypertonic NaCl. In addition, CBX produced sodium retention during sub-chronic NaCl load and sodium imbalance. Therefore, the integrity of the carotid bodies is important for the cardiovascular response to acute hypernatremia, as well as sodium balance during acute and sub-chronic NaCl exposure. The impairment of these mechanisms may contribute to physio-pathological disorders such as hypertension, cardiac failure, and edema, reinforcing the importance of osmosensory function of the carotid bodies.

## Author Contributions

All experiments were performed at the Federal University of Goiás. EdS, GP, and EC designed the experimental protocols. EdS, AdM, ELJ, KR, and LN performed the experiments. EdS, AdM, ELJ, AR, JM, GP, and EC analyzed the data. EdS, AdM, ELJ, KR, LN, WC, AR, AF-O, JM, GP, and EC interpreted the data, drafted, revised, and approved the final version of the manuscript.

## Conflict of Interest Statement

The authors declare that the research was conducted in the absence of any commercial or financial relationships that could be construed as a potential conflict of interest.

## Abbreviations

CBX: carotid body removal
CVLM: caudal ventrolateral medulla
HR: heart rate
KCN: potassium cyanide
MAP: mean arterial pressure
Na^+^: sodium
NTS: nucleus of the tract solitary
PAP: pulsatile arterial pressure
PVN: hypothalamic paraventricular nucleus
SON: supraoptic nuclei
